# Radial head subluxation in a chondrodystrophic cat: aetiology, surgical treatment and outcome

**DOI:** 10.1177/20551169251366438

**Published:** 2025-09-19

**Authors:** Katharina Lunde, Sorrel J Langley-Hobbs

**Affiliations:** 1 University of Bristol Faculty of Health Sciences, Langford Vets, Bristol, UK; 2 University of Bristol Faculty of Health Sciences, Veterinary School, Bristol, UK

**Keywords:** Angular limb deformity, radial head subluxation, orthopaedic surgery, ulnar osteotomy, Prolene toggle, chondrodystrophy, Dwelf, Munchkin, Sphynx, American Curl

## Abstract

**Case summary:**

A 10-month-old male neutered Dwelf cat with bilateral thoracic angular limb deformity secondary to chondrodystrophism presented with an acute onset of left thoracic lameness due to elbow subluxation and radial head displacement. Financial restrictions limited treatment to a single surgical procedure consisting of a left ulnar osteotomy, radioulnar polypropylene toggle placement and two temporary radioulnar transosseous K-wires. The lameness resolved but a degree of radial head subluxation persisted, radiographically similar in degree to the contralateral limb.

**Relevance and novel information:**

Chondrodystrophism is not common in cats. Inbreeding and chondrodystrophism is likely to result in the diagnosis of musculoskeletal diseases that have not previously been reported in this species. To the authors’ knowledge, this is the first case report of a developmental radial head subluxation in a chondrodystrophic cat.

## Introduction

Congenital and developmental elbow luxation or subluxation is uncommon in cats.^1^ Only three feline cases have been published previously.^2–4^ The terms ‘congenital’ and ‘developmental’ elbow luxation are often used interchangeably in the literature, as they share similar clinical presentations, radiographic findings and are frequently diagnosed late, making differentiation challenging.^5,6^ They are classified according to anatomy.^3,7^ Type 1 represents humeroradial luxation, type 2 represents humeroulnar luxation and type 3 occurs when both the ulna and radius are displaced.^3^ In dogs, type I elbow luxation is suspected to be developmental, as seen in chondrodystrophic dog breeds such as the Dachshund and Shih Tzu.^5,6,8^ These breeds have cubital varus, radial head subluxation and slow growth in the distal ulnar growth plate as features of their chondrodystrophism.^9,10^ The short ulna causes pressure at the radiohumeral joint, which can result in lateral radial head subluxation.^8^

## Case description

A 10-month-old male neutered Dwelf cat weighing 3.3 kg was referred for evaluation of a 3-month history of acute-onset left thoracic limb lameness and bilateral forelimb angular deformity, which had partially respo-nded to rest and anti-inflammatory medication. At presentation, the cat was bright, alert and responsive, with a left thoracic limb lameness of 6/10–10/10.^11^ Despite this, he was still able to jump, predominantly using the right thoracic limb. Bilateral abduction of the elbows was noted, and the left radial head was palpable luxated laterally. The right elbow had a reduced range of motion, with pain elicited on manipulation. Marked bilateral procurvatum of the antebrachium and carpal valgus were also present. Pelvic limb examination was unremarkable.

**Figure 1:**
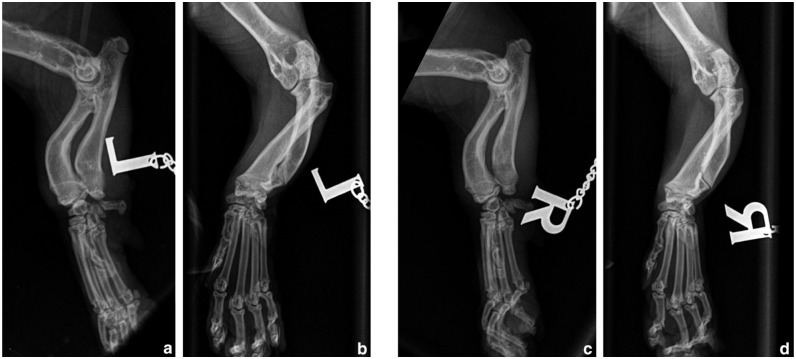
Mediolateral and craniocaudal radiographs of the left (a,c) and right (b,d) thoracic limbs. There is bilateral radial head subluxation, radial curvus, short ulna with premature closure of the distal ulnar growth plate and varus deviation of the ulna. The changes are compatible with chondrodystrophy, bilateral angular limb deformity, elbow dysplasia and subsequent developmental type I radial head subluxation, as well as humeroulnar subluxation, marginally worse on the left. *Courtesy of the referring clinic*

**Figure 2:**
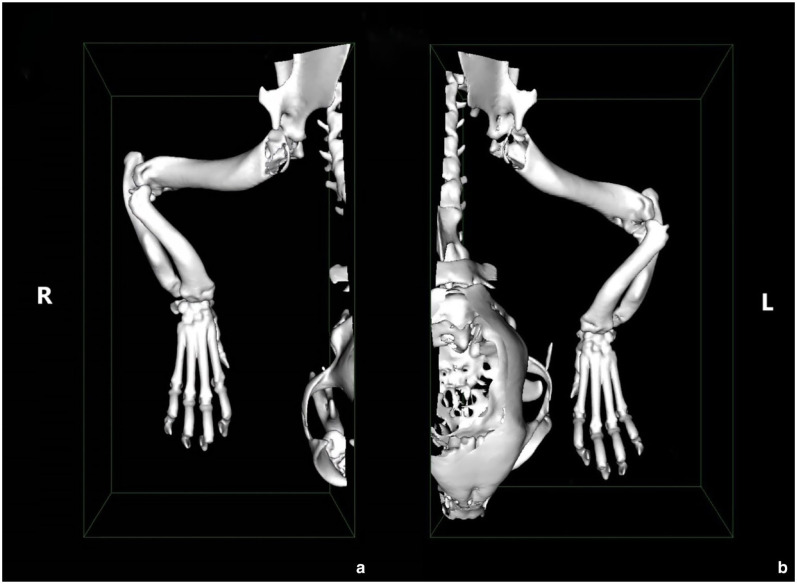
Dorsal view of the three-dimensional render volume of CT of the (a) right and (b) left thoracic limbs. These show bilateral forelimb angular deformity, including procurvatum of the radius, external torsion of the paw and carpal valgus. On the left side, there is a more marked subluxation of the radial head

Preoperative CT revealed bilateral thoracic limb angular deformities, including procurvatum of the radius, external torsion of the paw and carpal valgus. Bilateral elbow incongruity and radial head subluxation were documented, mild on the right and marked on the left (Figures 1 and 2). The diagnosis was bilateral thoracic limb angular deformity, presumed secondary to slow growth in the distal ulnar physis due to chondrodystrophism. The combination of radial head subluxation and humeroulnar subluxation of the left thoracic limb was presumed to be the cause of lameness. After discussing the findings with the owner, a single corrective surgery was planned with the aim of reducing the radial head subluxation and improving joint congruity. As a result of financial constraints, the owner would permit only one surgery.

Before surgery, the cat was premedicated with dexmedetomidine (10 µg/kg IM, Dexdomitor; Vetoquinol) and methadone (0.3 mg/kg IM, Methadyne; Jurox), induced with alfaxalone (0.9 mg/kg IV, Alfaxan; Zoetis) and maintained with 1.2% isoflurane (Isoflurane; Piramal Critical Care) in oxygen. Cefuroxime (20 mg/kg IV, Zinacef; Glaxo Smith Kline) was administered 30 mins preoperatively, repeated every 90 mins perioperatively and continued every 8 h postoperatively. Locoregional anaesthesia was provided with a brachial plexus block with bupivacaine (1 mg/kg, Marcain Polyamp Steripack; Aspen Pharma). A routine approach to the shaft of the ulna by skin incision over the lateral aspect of the proximal antebrachium^12^ and a single bioblique ulnar osteotomy was performed using a sagittal saw. Immediate reduction of the radial head was achieved, resulting in an immediate improvement in elbow joint range of motion. However, flexion of the elbow joint led to reluxation. To maintain reduction, a 2.0 mm polypropylene radioulnar toggle and button were placed by drilling a 1.1 mm bone tunnel across the radius and ulna with a battery-powered drill while the radial head was manually reduced. This improved the stability of the radial head, although some degree of subluxation persisted in flexion.

**Figure 3:**
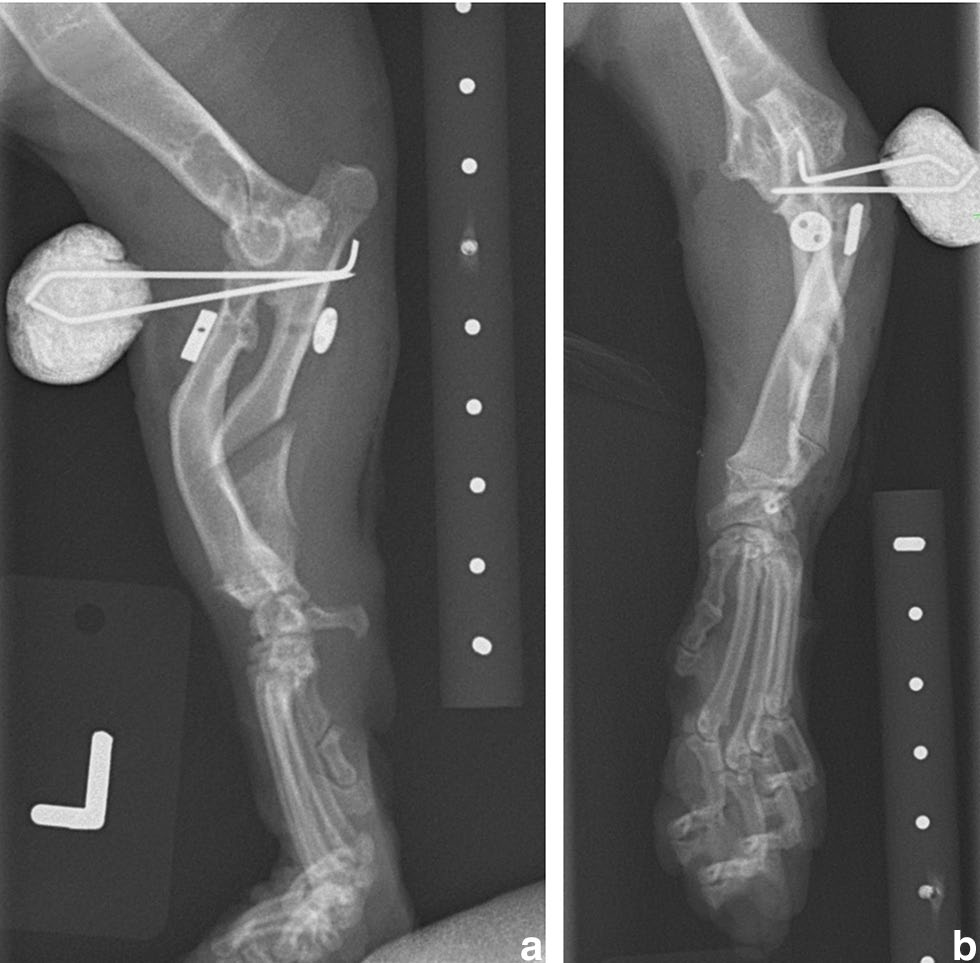
Immediate postoperative (a) mediolateral and (b) craniocaudal radiographs of the left thoracic limb. The radial head has been reduced into a more anatomical position. There is a gap at the ulnar osteotomy site compatible with release of the ulna and dynamic distraction. There is a radiolucent tunnel across the radius and ulna with a radioulnar toggle pin and button and two diverging K-wires placed in a transosseous position between the radius and ulna and exiting the cranial surface of the proximal antebrachium

Further stabilisation was achieved by placement of two diverging temporary 0.9 mm (20 G) radioulnar K-wires. These exited the cranial surface of the proximal antebrachium with one end protected with putty and the other bent to reduce pin migration. Routine closure was performed, including skin sutures^12^ and postoperative radiographs were obtained (Figure 3). The cat was hospitalised for 48 h, analgesia was provided with methadone (0.2 mg/kg q4h) and meloxicam (0.1 mg/kg SC, Metacam; Boehringer Ingelheim). In addition, gabapentin (15 mg/kg PO q12h, Gabapentin; Summit) was given as an anxiolytic and sedative. The following day, the cat was ambulating satisfactorily. After discharge, the cephalexin (20 mg/kg PO q12h, Rilexine; Virbac) was continued for 7 days, meloxicam (0.05 mg/kg PO q24h) for 14 days and gabapentin (5 mg/kg PO q8h) for 4 weeks.

**Figure 4:**
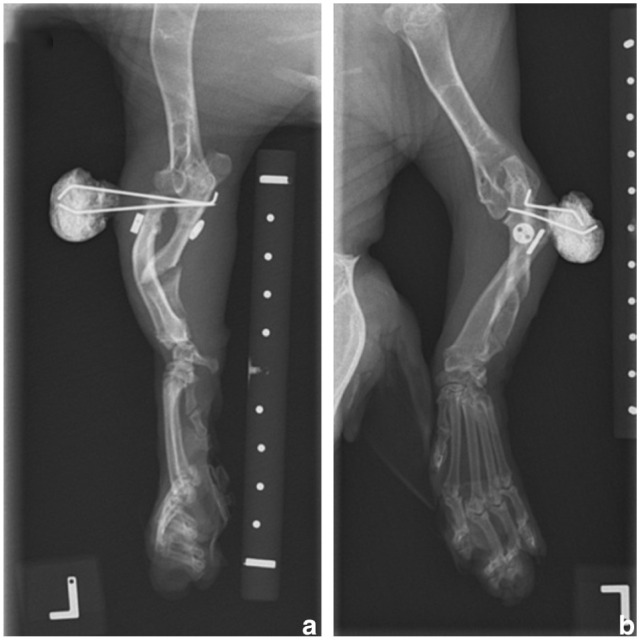
Three-week postoperative (a) mediolateral and (b) craniocaudal radiographs of the left thoracic limb. Implants are unchanged in position compared with the immediate postoperative radiographs (Figure 3). The radial head is still subluxated and the ulnar osteotomy site shows some remodelling

**Figure 5:**
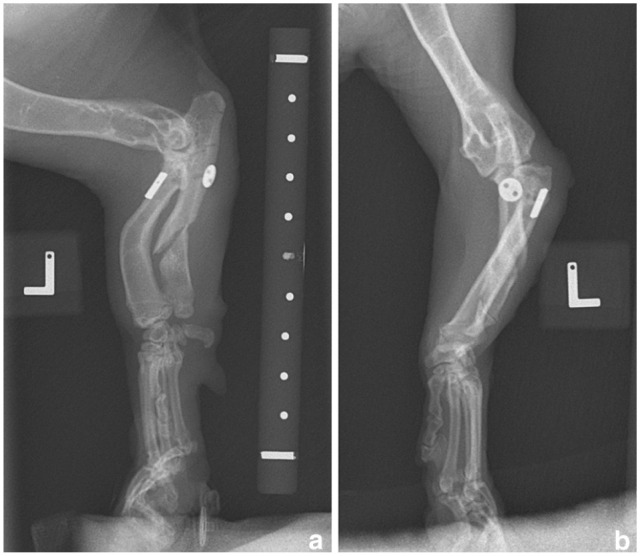
(a) Mediolateral and (b) craniocaudal radiographs of the left thoracic limb after removal of the temporary transosseous K-wires 3 weeks postoperatively. The radial head shows some degree of subluxation, subjectively in a similar position to its pre-pin removal state

On follow-up, 3 weeks postoperatively, the cat was bright, alert and responsive, with no reported complications. There was a left thoracic lameness score of 3/10,^11^ with no pain on palpation or flexion and extension of the elbow but a mild reduction in range of motion. Radiographs showed a mild persistent subluxation of the radial head and evidence of remodelling of the ulnar osteotomy site but no appreciable callus formation (Figure 4). A skin incision was made above the two diverging radioulnar K-wires and they were removed without complication. Postoperative radiographs were obtained to document implant removal (Figure 5). The cat was dispensed with meloxicam (0.05 mg/kg PO q24h) for 14 days and gabapentin (15 mg/kg PO q8h) for 4 weeks.

**Figure 6:**
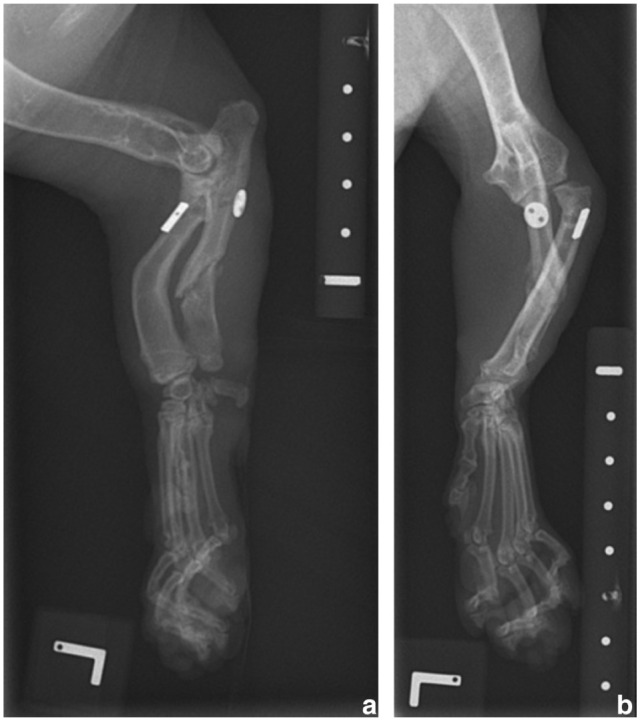
(a) Mediolateral and (b) craniocaudal radiographs of the left thoracic limb 6 weeks postoperatively. The toggle pin and button remain unchanged in position. A bony callus has developed and is starting to bridge the ulnar osteotomy gap. There is persistent lateral subluxation of the radial head

At 6 weeks postoperatively, the cat was returned for clinical assessment and radiography. The owner reported the cat to be ambulating well, and sitting with its left thoracic limb extended in front, with the elbow joint normally flexed, which it had not done since before the acute onset of lameness. Clinical examination revealed occasional lifting of the leg and a lameness score of 1/10.^11^ The radial head was subjectively less prominent upon palpation compared with its preoperative state, and there was a good range of motion in the left elbow. Radiographs obtained at this time (Figure 6) confirmed a similar position of the radial head subluxation to previous postoperative radiographs and evidence of healing of the osteotomy site. The cat was discharged with a rehabilitation plan and no medication (see Videos 1 and 2 in the supplementary material).

The cat was re-examined 5.5 months after the primary surgery after a short 48-h bout of lameness; however, there was no change on physical examination and at presentation the lameness had resolved and no further imaging was performed.

## Discussion

This case report is the first to document a developmental radial head subluxation as a cause of lameness in a chondrodystrophic cat. Chondrodystrophic dog breeds such as the Pekingese, Dachshund and Bulldog are over-represented in cases of radial head luxation, with proposed underlying mechanisms such as ulnar physeal injury, improper intraarticular annular ligation formation or hereditary factors.^8,13^ In cats, no breed over-representation has been identified.^2,4,14,15^ Feline disproportionate dwarfism is a chondrodystrophic condition similar to that in dogs, with similarities in phenotypes but different genetic aetiopathogenesis.^1^ Because of the suspected hereditary origin of these conditions resulting in angular limb deformity, reduced range of motion, change of limb kinetics and biomechanical loading likely to affect joint health and quality of life,^9,14,15^ careful consideration should be given when deliberately breeding chondrodystrophic animals.

The Dwelf is a cross between the Sphynx, Munchkin and American Curl, resulting in the main characteristic traits from the three breeds: hairlessness, chondrodysplastic traits (dwarfism) and curled ears.^11,13^ The Munchkin has a genetic mutation causing skeletal abnormalities similar to pseudoachondroplasia and appears prone to degenerative joint disease.^16^ The American Curl is characterised by curled ears, and although no skeletal abnormalities have been observed, the curled ears may be a manifestation of a more fundamental cartilage formation disturbance.^17^

Early surgical correction of developmental subluxation may offer a better prognosis than correction for congenital dislocation.^5,6,8^ Proposed surgical treatments for non-traumatic elbow luxation in cats and dogs include the radioulnar toggle and button technique,^18^ open reduction with extra-articular pin fixation and trans-articular external fixation,^19^ temporary transarticular pins,^8,20,21^ a combination of radial head ostectomy and radioulnar synostosis,^6^ radial osteotomy with external skeletal traction devide,^5^ arthrodesis of the elbow joint^7^ and radial head ostectomy.^3,22^ Three case reports describe congenital or developmental radial head luxation in cats. All three cats, two male and one female, were young (aged 7–9 months) and of European or unknown breed, with no mention of chondrodystrophic features. Two cases were bilateral, and surgical correction was not attempted because of the absence of functional impairment. The remaining case was unilateral and treated with radial head ostectomy. Short-term follow-up in all cases showed resolution of lameness and pain.^2–4^

A single oblique ulnar osteotomy was performed in the cat in this case report as it addressed the radial head and humeroulnar subluxation.^23^ The decision to add the radioulnar polyethylene suture and temporary transarticular stabilisation with two diverging K-wires was made to try and address the recurrent radial head luxation but still maintain some movement in supination and pronation based on a previously published case report in a dog.^18^ Cats have a greater degree of supination and pronation than dogs.^24^ The use of a radioulnar toggle has also been shown to be successful in the surgical correction of traumatic cranial luxation of the radial head in cats.^25^ Temporary external transarticular fixation has proven beneficial in the treatment of traumatic elbow luxation in cats.^26^ We considered applying an external fixator in this case, but because of the cat’s small size, the risk of radial head fracture was considered too high. Instead, diverging transosseus K-wires were placed. Prolonged immobilisation increases the risk for joint stiffness, muscle atrophy, reduction in range of motion and osteoarthritis.^26^ This influenced our decision to remove the diverging K-wires 3 weeks postoperatively, as leaving them in for too long would have increased the risk of fatigue breakage^26^ of the 0.9 mm pins.

Limited data are published on congenital and developmental elbow luxation cases managed either conservatively or surgically and long-term follow-up is lacking, especially in the former.^2–6,8,18–22,27^ In dogs, it has been reported that reduction of the radial head before the age of 5 months may allow remodelling of the articular cartilage.^23^ In this case, an acceptable clinical outcome was achieved with a decrease in elbow pain and thoracic limb lameness and an increase in elbow range of motion. Radiographs did not show a significant change in position of the radial head, and the ulnar osteotomy alone was probably the most important aspect of surgery in treating the cat’s elbow dysplasia and improving joint congruity.

## Conclusions

This is the first reported case of an acute developmental radial head subluxation in a chondrodystrophic cat. As type I elbow luxation is common in chondrodystrophic dogs, with the increasing popularity of chondrodystrophic cat breeds, the incidence of radial head luxation might increase in cats.^8,13,28^ As a result of the heterogen-eous presentation of congenital and developmental elbow luxation,^2–6,8,18–22,27^ and the limited published data, each case should be carefully assessed to ensure appropriate treatment.^12,20,25^
